# Isolation, characterization, and genomic analysis of a novel bacteriophage MA9V-1 infecting *Chryseobacterium indologenes*: a pathogen of *Panax notoginseng* root rot

**DOI:** 10.3389/fmicb.2023.1251211

**Published:** 2023-09-14

**Authors:** He Zou, Yafang Ding, Junjie Shang, Chunlan Ma, Jinhua Li, Ye Yang, Xiuming Cui, Jinhao Zhang, Guanghai Ji, Yunlin Wei

**Affiliations:** ^1^Faculty of Life Science and Technology, Kunming University of Science and Technology, Kunming, Yunnan, China; ^2^Key Laboratory of Sustainable Development and Utilization of Panax notoginseng Resources in Yunnan Province, Faculty of Life Science and Technology, Kunming University of Science and Technology, Kunming, Yunnan, China; ^3^State Key Laboratory for Conservation and Utilization of Bio-resources in Yunnan, Yunnan Agricultural University, Kunming, Yunnan, China

**Keywords:** *Panax notoginseng*, *Chryseobacterium indologenes*, root-rot, genome analysis, myovirus, phage therapy

## Abstract

*Chryseobacterium indologenes* is one of the primary causative agents of root rot of *Panax notoginseng*, which significantly affected plant growth and caused economic losses. With the increasing incidence of antibiotic-resistant bacterial phytopathogens, phage therapy has been garnered renewed attention in treating pathogenic bacteria. However, the therapeutic potential of phage therapy on root rot of *P. notoginseng* has not been evaluated. In this study, we isolated a novel lytic phage MA9V-1 infecting *C. indologenes* MA9 from sewage and monitored the formation of clear and round plaques with a diameter of approximately 0.5–1.5 mm. Phage MA9V-1 exhibited rapid absorption (>75% in 8 min), a latency period of 20 min, and a burst size of 10 particles per cell. Transmission electron microscopy indicated that the phage MA9V-1 is a new myovirus hosting *C. indologenes* MA9. Sequencing of phage genomes revealed that phage MA9V-1 contained a linear double-stranded DNA genome of 213,507 bp with 263 predicted open reading frames, including phage structure, host lysing, and DNA polymerase/helicase but no genes of tRNA, virulence, and antibiotic resistance. Our proteomic tree and genomic analysis revealed that phage MA9V-1 shares identity with *Sphingomonas* phage PAU and *Tenacibaculum* phage PTm1; however, they also showed apparent differences. Further systemic evaluation using phage therapy experiments on *P. notoginseng* suggested that phage MA9V-1 can be a potential candidate for effectively controlling *C. indologenes* MA9 infection. Thus, we have presented a novel approach to solving root rot in *P. notoginseng*.

## 1. Introduction

*Panax notoginseng* (Sanqi) is a famous traditional Chinese herb belonging to the family of Acanthopanax and is predominantly grown in Wenshan, Yunnan Province, China (Zhang et al., 2020a). *P. notoginseng* has become one of the most important crops in Yunnan Province owing to its unique medicinal efficacy and economic value and has been cultivated in China for over 400 years (Guo et al., 2010; Fan et al., 2016). The active pharmaceutical ingredients of *P. notoginseng* include saponins, brass, and polysaccharides. This herb has been characterized to have antitumor and antioxidant properties and has traditionally been used in treating cardiovascular diseases such as coronary heart disease (Yang et al., 2014; Xu et al., 2019). Similar to *P. ginseng*, the roots of *P. notoginseng* have been used as the raw material for over 400 medicinal products, such as Xuesaitong capsules, Yunnan Baiyao, and pain relieving powder (Yang et al., 2013). With the increasing demand for *P. notoginseng* products and the rapid development of modern Chinese medicine, there has been increased attention to *P. notoginseng*. However, *P. notoginseng* yield has been severely affected owing to replanting soils, long growth cycles, and various diseases, resulting in a significant reduction in harvest (Tan et al., 2017).

Approximately 70% of medicinal plants have the problem of continuous cropping obstacles, which were caused by varying degrees of phytopathogen infection (Zhao et al., 2016). Among them, root rot disease (RRD) is the most destructive soil-borne disease and is difficult to control using chemical pesticides or field management. One of the primary reasons hindering its eradication is the complexity of interaction between different pathogens, including bacteria, fungi, and parasitic nematodes (Jiang et al., 2011). Continuous cropping for *P. notoginseng* will increase root rot incidence, resulting in a 5%–20% average loss of yield, which may rise to over 70% in unfavorable situations and even with no harvest (Dong et al., 2016). Currently, most research studies on root rot disease have focused on aspects such as screening of antifungal pathogens (Li et al., 2022), the function of endophytic fungi (Zheng et al., 2017), and analysis of fungal community composition in soil (Wang et al., 2021). However, these studies have not yet been able to completely solve or alleviate *P. notoginseng* root rot. Notably, there are very few reports on the bacterial pathogens that infect *P. notoginseng* to date.

Zheng et al. (2022) isolated a bacterium *Stenotrophomonas maltophilia* that is capable of causing *P. notoginseng* root rot from rotten root samples. Similarly, *Chryseobacterium indologenes*, with yellow-orange colony, slightly viscous, and translucent, are aerobic, non-fermenting, catalase-positive gram-negative bacilli. It is known as rare human conditional pathogenic bacteria but rarely reported as a plant pathogen (Aykac et al., 2016). Until 2020, the team of Professor Ji isolated a strain *C. indologenes* MA9 that can also infect and result in *P. notoginseng* root rot, which was the first report indicating *C. indologenes MA9* as a plant pathogen to infect *P. notoginseng* in China (Zhang et al., 2020b). Currently, solutions to bacterial pathogens rely mainly on pesticides (Yang Y. et al., 2022) and antibiotics (Xie et al., 2018), which cause irreversible environmental pollution. Therefore, there is an urgent need for better methods to control these causative agents to improve crop yields and reduce economic losses.

Bacteriophages (phage) are the most abundant biological organisms on the earth and can be classified as lytic and lysogenic phages according to their living habits, which are crucial to maintaining the ecosystem balance (Din et al., 2016). Phages with the ability of high specificity to their hosts are considered an alternative solution to the crisis of antibiotic abuse and thus have been arisen much interests in many industries, especially in the agriculture. For example, RsoM1USA, a strain of *Ralstonia* phage screened from tomato fields in Florida, is a new species of *P2virus*, which can effectively slow down leaf wilting of tomato plants infected with *Ralstonia* when used with a multiplicity of infection (MOI) of 0.01 (Addy et al., 2019). Similarly, phage PHB09 against the plant pathogen *Pseudomonas syringae* pv*. actinidiae* causing kiwifruit bacterial canker exhibited a strong ability to kill host cells both *in vitro* and *in vivo*, which is expected to be a potential biocontrol agent in reduction of crop economic loss (Liu et al., 2021). In addition, a combination of various phages infecting the same host cell was used to prepare a phage cocktail that successfully and effectively suppressed the brown blotch disease of oyster mushrooms by killing the pathogen *Pseudomonas tolaasii* (Yun et al., 2022). However, the applicability of phage therapy on root rot of *P. notoginseng* has not been reported previously in China, and its therapeutic potential on root rot has not been evaluated.

In this study, we isolated and characterized the first *C. indologenes*-infecting phage MA9V-1 from sewage samples. We also compared the whole genome of phage MA9V-1 with others, determined that it is a new virus targeting *C. indologenes*, and evaluated its efficacy in preventing *P. notoginseng* root rot to provide a novel approach to solve this major challenge.

## 2. Materials and methods

### 2.1. Bacterial strain and growth conditions

*C. indologenes* strain MA9 (accession no. CP075170), isolated from rotten roots of *P. notoginseng*, was provided by professor Ji of Yunnan Agricultural University and was used as a host in this study. Samples for screening phages were collected from different regions as follows: (1) The rhizospheric soils of healthy and rotten *P. notoginseng* were collected from Pingyuan Street, Yanshan County, Wenshan Prefecture, Yunnan Province in November 2020; (2) the rotten roots of *P. notoginseng* and *P. notoginseng* root soils were collected from Shilin Yi Autonomous Region, Kunming City, Yunnan Province in January 2021; (3) the soils of different vegetables and fruit trees were collected from the farmland near Kunming University of Science and Technology, Chenggong District, Kunming City, Yunnan Province in January 2021; and (4) tewage samples were collected from the First People’s Hospital of Yunnan Province in February 2021. *C. indologenes* MA9 was cultured in an optimized nutrient agar (NA) medium (10 g peptone, 5 g sodium chloride, and 3 g beef extract) at 160 rpm at 28°C.

### 2.2. Isolation, purification, and propagation of phages

First, we tried to isolate phages from the rhizospheric soil mixtures of healthy and decayed *P. notoginseng* infected with *C. indologenes* MA9; however, we could not find any phage. Subsequently, under the same culture conditions, we attempted to use other samples such as rotten roots, *P. notoginseng* root soils, and soils of various fruits and vegetables. However, the targeted phage was also not found. Finally, we isolated a *C. indologenes* strain MA9 phage from sewage samples of the First People’s Hospital of Yunnan Province, China, using the virus enrichment and double-layer agar plate methods (Pajunen et al., 2000). In brief, samples for phage isolation were filtered through a 0.22-μm filter; then 50 mL filtrate was added to a 250 mL conical flask containing 50 mL SM buffer (100 mM NaCl, 10 mM MgSO_4_, 50 mM Tris–HCl at pH 7.5, and 0.01% gelatin) and kept static for 12 h. Subsequently, 40 mL suspension, 30 mL of SM buffer, 25 mL of NA liquid medium, and 2 mL of *C. indologenes* MA9 cultures were mixed and incubated overnight on a horizontal rotary shaker (160 rpm) at 28°C. The mixture was transferred to a sterile 50 mL centrifuge tube and centrifuged at 15,000 × g for 15 min at 4°C. Subsequently, the supernatant was filtered with 0.22-μm microporous membranes. In total, 5 mL filtrate was taken and mixed with 50 mL *C. indologenes* MA9 host cell cultures during the period of exponential growth phase [optical density at 600 nm (OD_600_) =0.6–0.8] and then repeated 2–3 times to enrich phage MA9V-1. Successively, 200 μL of phage stock was serially diluted with SM buffer, and different dilutions were mixed with 300 μL of *C. indologenes* MA9, which were added to 4.5 mL semi-solid NA medium after being incubated at 28°C for 20 min, and finally, the solution was finally poured onto the surface of the NA solid medium. A single clear plaque was picked from NA double-layer agar plate incubated overnight at 28° C, placed into 1 mL of SM buffer, and vortexed vigorously before filtering through a 0.22-μm membrane. The above steps were repeated 2–3 times to obtain pure phage MA9V-1 particles, and the phage MA9V-1 titer was measured to be 10^9^ PFU/ml by the double-layer agar plate method. The isolates were stored at 4°C.

### 2.3. Designing the optimal multiplicity of infection

The optimal multiplicity of infection (MOI) is the ratio of phages to bacteria at the time of infection. The experimental method followed was according to the study mentioned in the reference (Sun et al., 2022). In brief, the CFU/ml of the host cell at the exponential growth phase was counted through the dilution coating method with serial dilutions of 10^−4^, 10^−5^, and 10^−6^. Phage stock of known titer and host cells at the exponential stage were mixed with a gradient of MOI ranging from 0.001 to 10 and cultured in a shaker (180 rpm/min) at 28°C for 12 h. The enrichment was centrifuged at 15,000 × g at 4°C for 15 min and filtered through a 0.22-μm filter membrane, and the phage titer was determined by the double-layer agar plate method with three replicates.

### 2.4. Phage adsorption assay

Adsorption rate, a value of phage particles adsorbed to the host cell at a particular time, was determined with minor modifications, as previously described (Sasikala and Srinivasan, 2016). In brief, 9 mL of *C. indologenes* MA9 cultures with absorbance of OD_600_ at 0.6–0.8 was infected with 1 mL phage dilution (10^6^ PFU/ml) at multiplicity of infection of 0.01 and transferred to a sterile 50 mL conical flask to incubate at 28°C. Approximately 100 μL of culture was taken out at 4-min intervals (0, 4, 8, 12, 16, and 20 min) and added to sterile 2 mL tubes with 1.9 mL of pre-cooled NA medium, vortexed for 10–15 s, and then centrifuged at 15000 × g at 4°C for 15 min, to remove the cell-phage complexes. The titer of free phage in the supernatant was determined by the double-layer overlay method and was repeated three times (Stuer-Lauridsen et al., 2003).

### 2.5. One-step growth curve

To determine the parameters of the one-step growth curve, the protocol, as described earlier, was adopted (Wu et al., 2007) and modified according to the characteristics of the phage. *C. indologenes* MA9 was cultured up to the exponential growth phase (10^8^ CFU/mL), and 10 mL of culture was collected by centrifugation (11,000 × g, 8 min, 4°C) for harvesting, and the precipitate was resuspended with 2 mL of fresh NA liquid medium. Phage was added to the suspension at MOI = 0.01 and incubated at 28°C for 15 min without shaking. The mixture was, then, centrifuged at 11,000 × g for 8 min to remove the free phage particles, and the pellet was suspended with fresh NA medium to culture in a shaker at 180 rpm at 28°C after washed twice with 10 mL nutrient agar medium. In total, 200 μL of culture was taken out every 10 min until 130 min and mixed with 800 μL SM buffer. Finally, the mixture was centrifuged at 15,000 × g for 3 min at 4°C. The titers of the samples were immediately measured by the double-layer agar method. The burst size was calculated according to the following definition: Burst size is the ratio between the number of phage particles at the end of lysis and the number of the host cells at the beginning of the infection (Li et al., 2021). All assays were repeated in three independent experiments.

### 2.6. Thermal and pH stability

For determining the thermal and pH stability of phage MA9V-1, the experimental protocol was performed as proposed with slight modification (Sasikala and Srinivasan, 2016; Sun et al., 2022). In brief, 1 mL phage MA9V-1 lysate (10^10^ PFU/m) was treated under different temperatures from 30 to75°C at 5°C intervals for 1 h. Then, these treated samples checked the survival rate of phage MA9V-1 using the double-layer agar assay. Similarly, 100 μL of phage stock (10^10^ PFU/ml) was added to 900 μL different buffers of pH ranging from 3 to11 (component of citrate sodium, potassium dihydrogen phosphate, Tris–HCl, and sodium carbonate) for incubation of 1 h at 30°C. After treatment, the titer of phage MA9V-1 was evaluated using the same procedure. All assays were performed in triplicate.

### 2.7. Phage host range

The host range of *Chryseobacterium* phage MA9V-1 was tested against 11 different bacterial strains using the standard spot testing assay, including *Chryseobacterium indologenes* (*n* = 7), *Bacillus cereus* (*n* = 1), *Pseudomonas syringae* (*n* = 1), and *Escherichia coli* (*n* = 1), of which *Chryseobacterium indologenes* ATCC 29897, *Pseudomonas syringae* CGMCC 1.3070, and *Escherichia coli* ATCC 11303 were type strains purchased from Strain Preservation Center, while the others were isolates. Each 300 μL of bacterial cells was mixed with 200 μL of 10-fold series gradient dilutions (10^−1^–10^−7^) of phage MA9V-1 suspension (10^10^ PFU/ml), then adding all mixtures to 4.5 mL semi-solid medium before pouring into the surface of double-layer agar plates, respectively, and incubated at 28°C overnight in triplicate.

### 2.8. Transmission electron microscopy

The morphology and structure of pure phage particles were visualized using transmission electron microscopy (TEM). The phage particles purified with the density gradient centrifugation method were loaded on a carbon-coated copper grid to adsorb for 10 min, followed by negative staining with 1% phosphotungstic acid. The stained phage MA9V-1 was observed using transmission electron microscopy (Hitachi HT7820) at 120 kV (Johnson et al., 2011).

### 2.9. Phage DNA extraction, sequencing, and bioinformatics analysis

To improve the purity of the phage genome, 2.5 μL DNase I (1 U/μl) and 0.5 μL RNase A (1 mg/mL) were added to 500 μL phage stock to remove the host cell nucleic acid, and further experimental operation was performed using the phenol–chloroform protocol, as previously described in the study mentioned in the reference (Chen et al., 2019). Nucleic acid concentration of phage was determined by NanoDrop (Shanghai Baoyude Scientific Instruments Co., Ltd., BIO-DL, Shanghai) and imaged by 1% agarose gel electrophoresis. Sequencing of the phage genome was performed by the Illumina_Novaseq_PE150 platform (Shenzhen Huada Intelligent Manufacturing Technology Co., Ltd., Illumina, Shenzhen). First, phage genome was randomly broken into approximately 300–500 bp fragments using the Covaris M220 (Shanghai Tusen Vision Technology Co., Ltd., Shanghai, China) DNA fragmentation instrument, discarding adapters and trimming sequences with a length of less than 50 bp. Then, using the BBMap version 38.51 (Roy and Chanfreau, 2020),^1^ comparisons were performed with the existing NCBI database to sift out the corresponding rRNA, host, and other bacterial genome sequences, to purify the phage genome. The assembly of clean reads was performed using the “*De novo* assembly” option of SPAdes v3.5.0 software (Prjibelski et al., 2020).^2^ Subsequently, contigs assembled with reads were comparatively analyzed with BLAST (v2.10.0+)^3^ (Camacho et al., 2009) to virus-NT, NCBI nt, and viral RefSeq databases to match the most similar gene reference sequences. Finally, the assembled phage MA9V-1 genome was annotated using Proksee (Grant and Stothard, 2008).^4^ Functional protein bioinformatic annotation of ORFs was predicted manually using HHpred (Soding et al., 2005).^5^ The virulence-associated and antibiotic resistance gene potentially existing in the phage MA9V-1 genome was analyzed through the virulence factor database (Liu et al., 2019)^6^ and the comprehensive antimicrobial research database.^7^ The presence of transfer RNA genes was assessed using tRNAscan-SE (Lowe and Chan, 2016).^8^ The phage MA9V-1 genomic data were aligned by Clustalw v2.1 (Peng et al., 2022)^9^ and analyzed using the visualization bioinformatics software Easyfig v2.2 (Peng et al., 2022).^10^ MEGA v11 (Tamura et al., 2021)^11^ was used to construct a phylogenetic tree for determining phylogenetic relationships between phage MA9V-1 and other strains. Furthermore, phage proteome phylogenetic tree was constructed by viral proteomic tree (ViPTree) server v1.9 (Nishimura et al., 2017).^12^

### 2.10. Characterization of phage structural proteins

Approximately 7 mL of phage particle suspension with titer of 10^9^ PFU/ml purified by the polyethylene glycol (PEG) precipitation method was ultracentrifuged with CsCl density gradient centrifugation (HIMAC CP-100WX Ultracentrifuge, HITACHI, Japan) at 4°C at 150,000 × g for 8 h and extracted the separated layer of phage with a sterilized syringe. Then, 20 μL of ultra-purified phage particles was mixed with 2× SDS loading buffer, heated in boiling water for 15 min, and subjected to SDS-PAGE [12% (w/v) polyacrylamide] (Uchiyama et al., 2014).

### 2.11. Virulence assay in *P. notoginseng*

The experimental material was the root of a healthy 2-year-old *P. notoginseng* plant collected from Shilin Yi Autonomous Region, Kunming City, Yunnan Province. After a series of pre-treatments, the slices of *P. notoginseng* root were placed onto a plate covered with wetted filter paper. In total, 1 mL of 24-h cultures of *C. indologenes* strain MA9 was transferred to a 50 mL conical flask and grown at 28°C with shaking until the culture reached the OD_600_ of 0.6–0.8. Phage MA9V-1 and *C. indologenes* MA9 were mixed with optimal multiplicity of infection (MOI) of 0.001, 0.01, 0.1, 1, and 10, respectively, and incubated at 28°C for 10 min. In total, 100 μL of different MOI mixtures were was added to the root slices, sealed with tape, and cultured in an incubator at 26°C. This experiment was divided into seven groups with eight parallels in each group. Among them, the negative control was the *C. indologenes* MA9 reaching the exponential stage, whereas the positive control was the inactivated *C. indologenes* MA9 at the log phase after treating with the autoclave sterilization method. Under the same experimental condition, 100 μL of the mixture of different MOI was added to the experimental group at 24-h intervals, the negative control and positive control were added to the equal volume of treatment liquid, and the number of infected and uninfected *P. notoginseng* plants was counted after 7 days. In this study, one condition for determining root rot was the rotting and softening of the root interior. The disease incidence was calculated as the percentage of rotten roots to the total number of parallel experiments. The experiment was performed in triplicate.

## 3. Results

### 3.1. Morphology of phage MA9V-1

**Figure 1:**
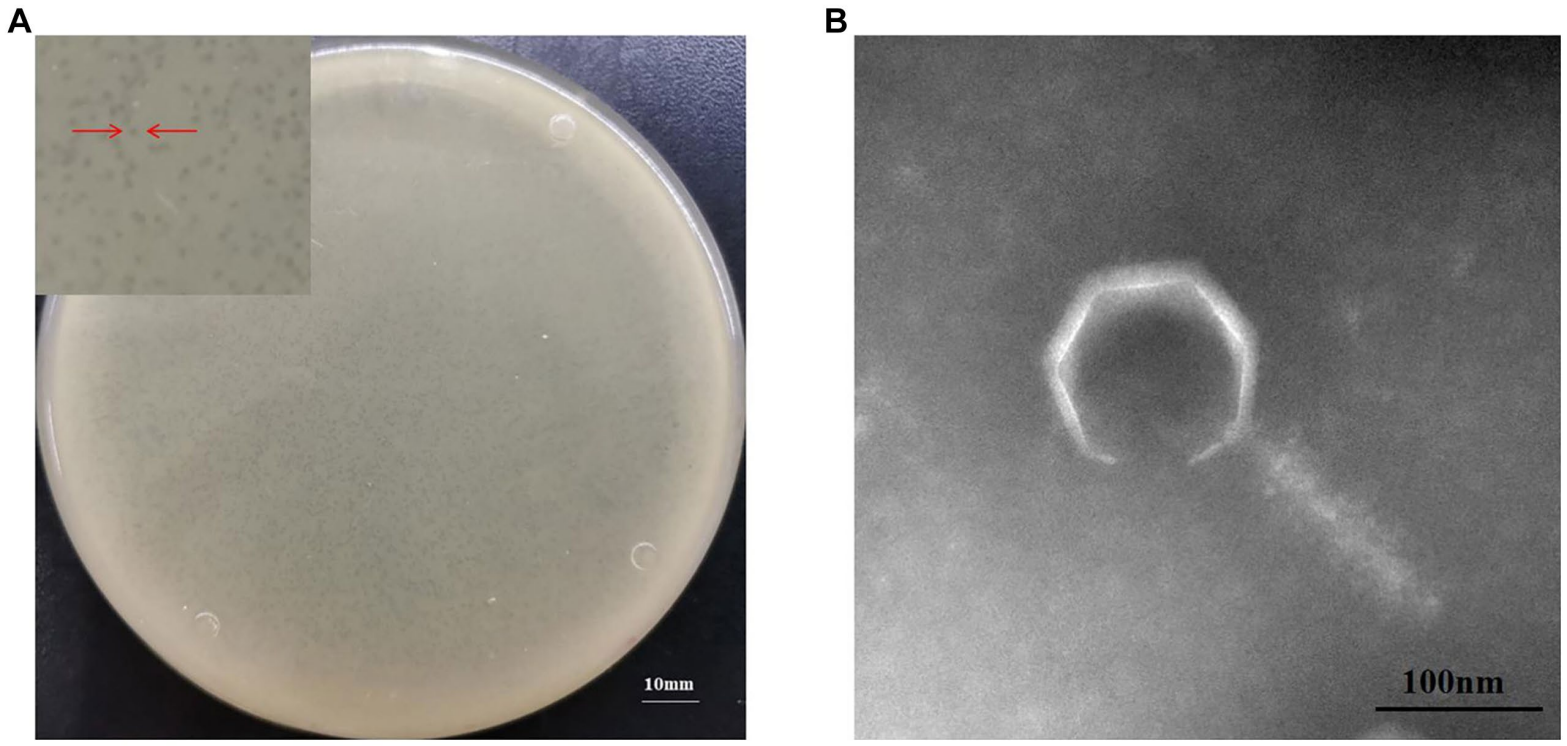
Morphological characteristics of *Chryseobacterium indologenes* phage MA9V-1. **(A)** Phage plaque morphology of phage MA9V-1 on an NA double-layer agar plate and **(B)** TEM image of phage MA9V-1.

To the best of our knowledge, phage MA9V-1 (formal name: vB_CinP_MA9V-1) of the *C. indologenes* strain MA9 is the first phage to have bacteriolytic activity against *C. indologenes* MA9. The results suggested that the phage MA9V-1 can form clear and round plaques with a diameter of approximately 0.5–1.5 mm on lawns of *C. indologenes* MA9 of the double-layered plate (Figure 1A). The morphological characteristics of phage MA9V-1 (Figure 1B) revealed that it has an icositetrahedron-coated protein “head” with a diameter of approximately 121.26 nm and a contractible tail length of approximately 170.34 nm with a width of approximately 44.62 nm. In addition, it also has a relatively short “neck” compared with other myoviruses such as *Ralstonia* phage RsoM1USA, a novel species of *P2virus* (Addy et al., 2019).

### 3.2. Biological characteristics of phage MA9V-1

**Figure 2:**
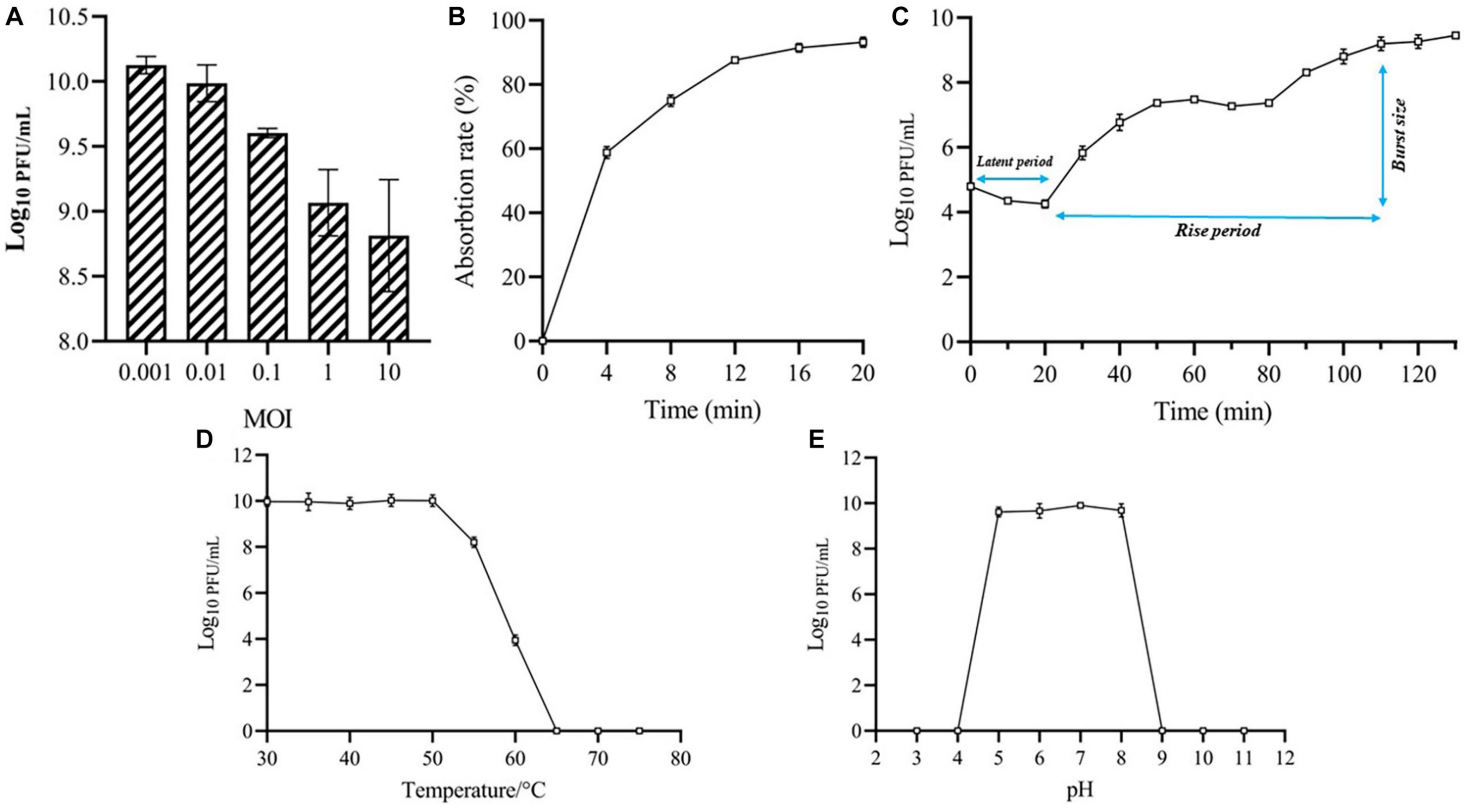
Biological features of phage MA9V-1. **(A)** Titers at different MOIs; **(B)** Adsorption curve; **(C)** One-step growth curve; **(D)** Thermal stability analysis; **(E)** pH stability analysis.

As shown in Figure 2A, phage MA9V-1 had the highest titer and was 1.25 × 10^10^ PFU/ml at an MOI of 0.001. The absorption curve revealed that phage MA9V-1 was capable of rapidly adsorbing onto the surface of the host cell membrane. Within 8 min, >75% of the phage particles had adsorbed, and after 12 min, approximately 90% were adsorbed, indicating that phage MA9V-1 had the ability of high adsorption efficiency (Figure 2B). The latent period, rise period, and burst size are three critical parameters to characterize the phage lifecycle; the results of the one-step growth curve suggested that these values are 20 min, 90 min, and 10 PFU/cell, respectively (Figure 2C). In the thermal stability experiment, Figure 2D shows that the temperature between 30°C and 50°C had no negative effect on phage MA9V-1 after a 1-h incubation. Even at 55°C and 60°C, the phage MA9V-1 also has the ability to infect *C. indologenes* MA9, but its viability is decreased to approximately 85 and 40% at 30°C, respectively. The infectivity of phage MA9V-1 was completely lost at 65°C and subsequent temperature of 70 and 75°C. Meanwhile, phage MA9V-1 had nearly no obvious variation by treating under different pH levels at 5 to 8 but showed strong sensitivity under extreme pH conditions and will be inactivated to no infectivity (Figure 2E). In addition, of 11 different bacterial cells examined, only 3 *C. indologenes* strains were found to be sensitive to the phage MA9V-1, namely *Chryseobacterium indologenes* MA9, *Chryseobacterium indologenes* 02, and *Chryseobacterium indologenes* 06, respectively (Table 1). When *Chryseobacterium indologenes* MA9 was considered as the host strain, phage MA9V-1 had the highest titer approximately two times than others.

**Table 1:** Host ranges of phage MA9V-1.

Strain	Lytic activity
*Chryseobacterium indologenes* MA9 (primary host)	**++**
*Chryseobacterium indologenes* 01	−
*Chryseobacterium indologenes* 02	**+**
*Chryseobacterium indologenes* 03	−
*Chryseobacterium indologenes* 04	−
*Chryseobacterium indologenes* 05	−
*Chryseobacterium indologenes* 06	**+**
*Chryseobacterium indologenes* ATCC 29897	−
*Bacillus cereus* MYB41-22	−
*Pseudomonas syringae* CGMCC 1.3070	−
*Escherichia coli* ATCC 11303	−

“++”: phage was efficient to infect host; “+”: phage was little efficient to infect host; “−”: phage was not to infect hot.

### 3.3. Genomic features of phage MA9V-1

**Figure 3:**
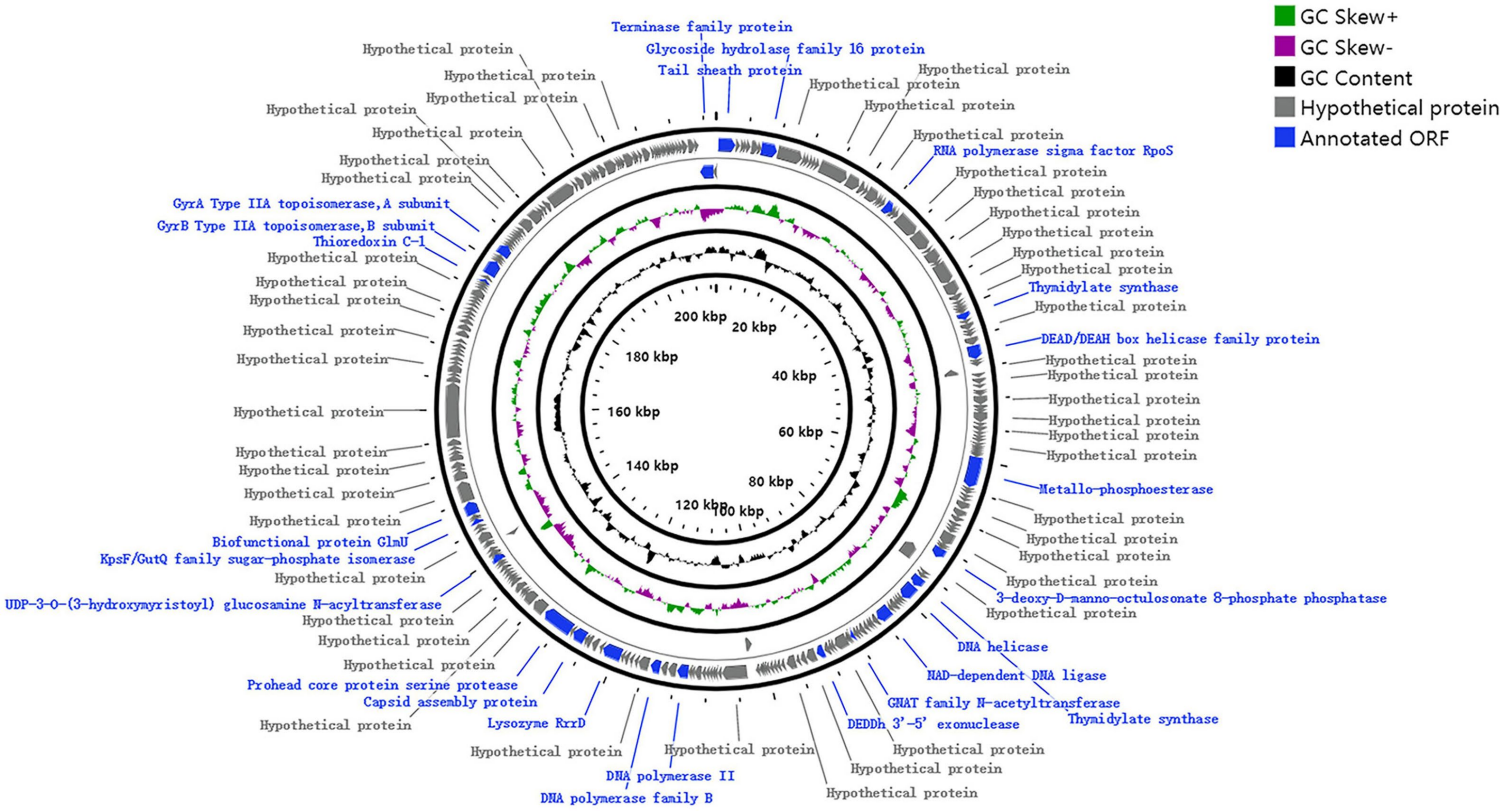
Whole-genome map of phage MA9V-1.

Phage genomic analysis is crucial to determine the specific functional proteins in the genome and the safety of phage application. The results showed that the genome sequence of phage MA9V-1, a linear double-stranded DNA (dsDNA), was 213,507 bp in length and contained 35.99% of G + C content (Figure 3). The phage MA9V-1 genome contained 263 putative open reading frames (ORFs); among them, 257 and 6 ORFs were identified in the forward and reverse strands, respectively (Table 2). The lengths of the longest and shortest ORF gene sequences were 7,122 bp and 90 bp, encoding one hypothetical protein with 2,374 amino acids and one hypothetical protein with 30 amino acids, respectively. According to the BLAST results, 39 ORFs had homologs to genes encoding known functional proteins, and 14 of these clustered into 3 modules related to aspects such as phage structure, host lysing, and DNA replication, and the remaining 25 genes encoded other biofunctional proteins. The absence of integrase in the genome indicated that phage MA9V-1 was a virulent phage targeting *C. indologenes* MA9 not a lysogenic phage. In addition, concerned tRNA gene, lysogenic gene, drug resistance gene, and virulent genes were not found in the phage MA9V-1 genome, indicating that the phage MA9V-1 depended on host translation and satisfied several recommended criteria for safe usage in the prevention and control of *C. indologenes* MA9 (Bruessow, 2012; Han et al., 2017).

**Table 2:** Annotated ORFs in the genome of phage MA9V-1.

ORFs	Strand	Start	Stop	Length	Function	Best-match	*E*-values	Per.ident	GenBank accession. no
ORF1	**+**	276	2,453	2,178	Phage tail sheath protein	*Tenacibaculum phage PTm1*	5e-38	31.95%	YP_009873700.1
ORF8	**+**	5,806	7,887	2082	Glycoside hydrolase family 16 protein	*Chryseobacterium aquifrigidense*	0.0	80.27%	WP_142018695.1
ORF16	**+**	17,616	19,355	1840	Short tail fiber protein	*Tenacibaculum phage pT24*	6e-18	25.67%	YP_009876786.1
ORF23	**+**	23,524	24,687	1,164	RNA polymerase sigma factor RpoS	*Ignavibacteriales bacterium*	3e-11	28.06%	MCF8305946.1
ORF26	**+**	25,112	25,681	570	CYTH domain-containing protein	*Synechococcus* sp. *A15-127*	9e-08	29.38%	WP_102469181.1
ORF40	**+**	40,762	41,706	945	Thymidylate synthase	*Flavobacterium stagni*	4e-116	55.95%	WP_129461710.1
ORF46	**+**	44,076	45,067	990	DEAD*/*DEAH box helicase family protein	*Tenacibaculum phage PTm1*	3e-28	25.47%	YP_009873724.1
ORF70	**+**	59,570	63,550	3,981	Metallo-phosphoesterase	*Sphingomonas phage PAU*	1e-66	24.59%	YP_007006895.1
ORF76	**+**	66,665	67,465	801	Ribonucleoside-diphosphate reductase subunit beta	*Amniculibacterium* sp. *G2-70*	8e-147	64.13%	WP_187477617.1
ORF77	**+**	67,632	68,348	717	Intron associated endonuclease	*CrAss-like virus* sp.	1e-11	43.52%	DAF35514.1
ORF84	**+**	72,188	73,558	1,371	3-deoxy-D-manno-octulosonate 8-phosphate phosphatase	*Sphingomonas phage PAU*	6e-10	34.29%	YP_007006823.1
ORF87	**+**	76,225	76,830	606	Thymidylate kinase	None	N/A	N/A	N/A
ORF88	**+**	76,818	78,161	1,344	Thymidylate synthase	*Butyrivibrio* sp.	8e-23	33.76%	MBQ7428460.1
ORF89	**+**	78,229	80,271	2043	DNA helicase	*Tenacibaculum phage PTm1*	4e-59	28.50%	YP_009873805.1
ORF95	**+**	82,582	84,492	1911	NAD-dependent DNA ligase	*Sphingomonas phage PAU*	7e-44	29.22%	YP_007006813.1
ORF103	**+**	88,184	88,636	453	GNAT family N-acetyltransferase	*Maritalea porphyrae*	2e-07	35.87%	WP_269392588.1
ORF110	**+**	92,533	93,522	990	DEDDh 3′-5′ exonuclease	*Citromicrobium phage vB_CbaS-RXM*	7e-15	31.46%	USM11553.1
ORF112	**+**	94,972	95,745	774	Baseplate protein	*Tenacibaculum phage pT24*	3e-29	33.73%	YP_009876769.1
ORF115	**+**	97,929	98,273	345	Septal ring lytic transglycosylase RlpA family protein	*Moraxella lacunata*	3e-26	52.73%	WP_115004602.1
ORF124	−	101,658	102,536	879	Neck protein	*Bacteriophage* sp.	1e-23	28.03%	DAV36558.1
ORF135	**+**	110,343	111,830	1,488	DNA polymerase II	*Ackermannviridae* sp.	2e-51	31.31%	DAM31303.1
ORF138	**+**	114,019	115,296	1,278	DNA polymerase family B	*Ackermannviridae* sp.	2e-51	31.31%	DAM31303.1
ORF144	**+**	119,283	121,751	2,469	Lysozyme RrrD	*Synechococcales cyanobacterium M58_A2018_015*	1e-31	45.64%	MBF2003654.1
ORF145	**+**	121,762	122,259	498	Head completion protein	*Tenacibaculum phage PTm1*	4e-26	43.87%	YP_009873685.1
ORF147	**+**	123,439	124,197	759	DNA end protector protein	*Caudoviricetes* sp.	1e-58	40.97%	DAP95445.1
ORF149	**+**	124,423	126,135	1713	Capsid assembly protein	*Caudoviricetes* sp.	2e-69	30.33%	DAQ63115.1
ORF151	**+**	126,501	130,544	4,044	Prohead core protein serine protease	*Caudoviricetes sp*	5e-23	39.46%	DAP95449.1
ORF165	**+**	139,385	140,299	915	UDP-3-O-(3-hydroxymyristoyl) glucosamine N-acyltransferase	*Caudoviricetes* sp.	7e-41	34.26%	DAN50236.1
ORF174	**+**	144,273	144,869	597	Dihydrofolate reductase	*Comamonadaceae bacterium*	7e-24	42.48%	RYX96028.1
ORF175	**+**	144,937	145,599	663	KpsF/GutQ family sugar-phosphate isomerase	*Chlamydia poikilotherma*	2e-25	40.99%	WP_117273901.1
ORF178	**+**	146,220	147,962	1743	Biofunctional protein GlmU	*Caudoviricetes* sp.	1e-46	27.38%	DAP95447.1
ORF206	**+**	175,015	176,130	1,116	Pyrimidine dimer DNA glycosylase/endonuclease V	*Unclassified Vibrio*	9e-08	29.38%	WP_102469181.1
ORF210	**+**	177,196	177,474	279	Thioredoxin C-1	*Flavobacterium* sp.	2e-16	39.33%	MCU0351479.1
ORF212	**+**	178,275	180,254	1980	GyrB Type IIA topoisomerase (DNA gyrase/topo II, topoisomerase IV), B subunit	*Uncultured Caudovirales phage*	1e-117	37.38%	CAB4159554.1
ORF216	**+**	181,261	182,730	1,470	GyrA Type IIA topoisomerase (DNA gyrase/topo II, topoisomerase IV), A subunit	*Uncultured Caudovirales phage*	2e-94	39.47%	CAB4219051.1
ORF232	**+**	194,744	195,652	195	CapA family protein	*Bacteroidota bacterium*	4e-18	31.45%	MBU2445609.1
ORF233	**+**	195,754	196,878	1,125	Hypothetical protein	*Virus NIOZ-UU157*	4e-51	55.70%	QPI16264.1
ORF237	**+**	197,909	198,853	945	Clamp loader of DNA polymerase	*Sphingomonas phage PAU*	4e-63	36.13%	YP_007006683.1
ORF246	**+**	203,955	204,944	990	Calcineurin-like phosphoesterase superfamily domain protein	*Uncultured Caudov* C*irales phage*	1e-126	54.88%	CAB5218431.1
ORF262	−	211,176	213,119	1944	Terminase family protein	*Sphingomonas phage PAU*	1e-77	29.01%	YP_007006694.1

### 3.4. Proteomic tree and comparative genomic analysis

**Figure 4:**
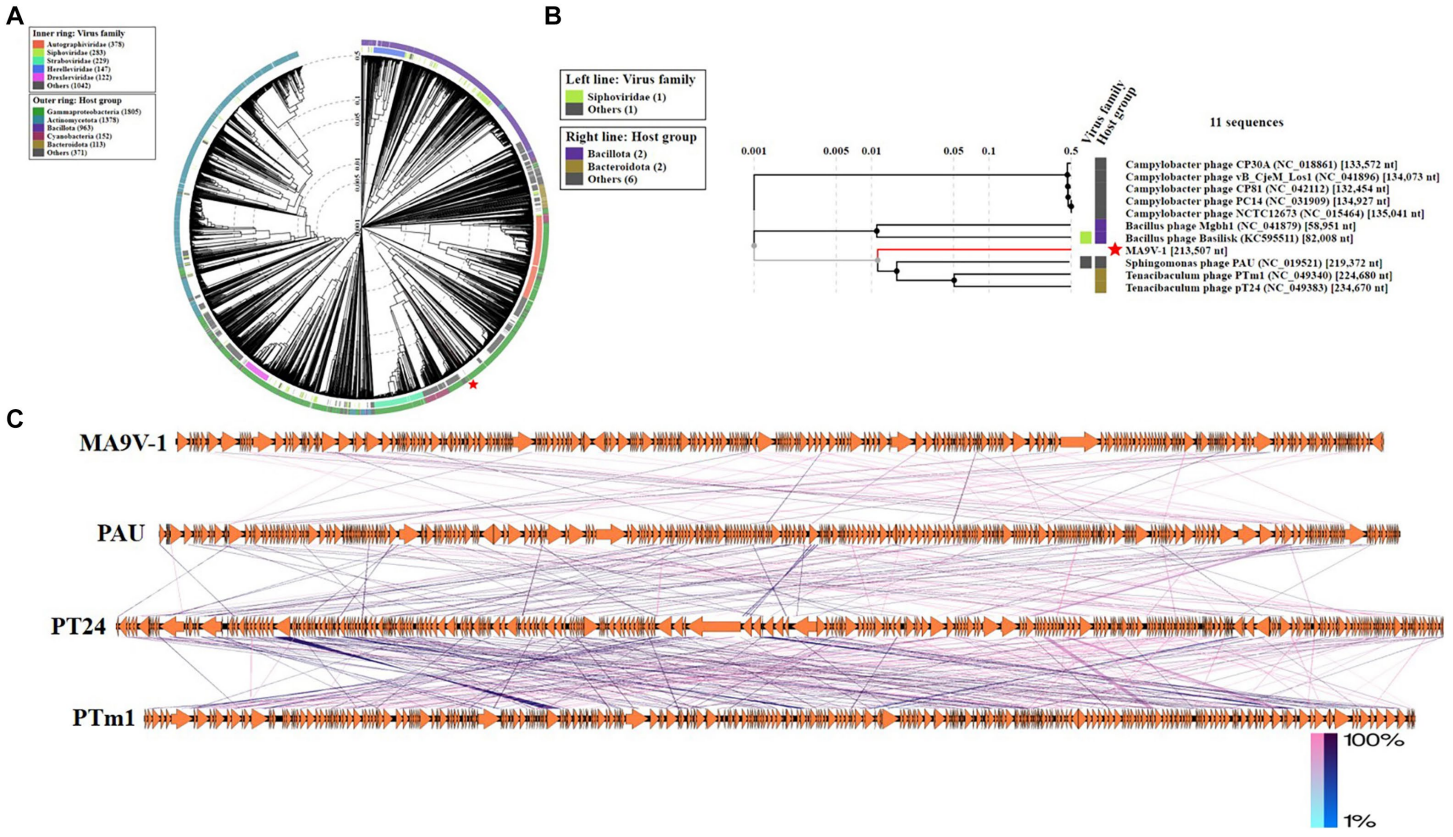
Proteomic tree of phage MA9V-1 generated using ViPTree. **(A)** A circular proteomic tree made with phage MA9V-1 and related prokaryotic dsDNA viruses. **(B)** The part of the rectangular proteomic tree of the enlarged part of area MA9V-1 of **(A)**. **(C)** Comparison of the draft genome sequence of *C. indologenes* phage MA9V-1(top) with other six homologous phages using EasyFig v2.2.5. The arrows of different colors indicate predicted ORFs and the direction of transcription. The color intensity from blue to red represents the level of amino acid sequence identity (1%–100%). The position of phage MA9V-1 is marked with a red “pentagram” star.

The phage MA9V-1 proteome is comprehensively analyzed through the ViPtree database and selected related genomes, which contain 2,201 virus families and 4,782 host groups. As shown in Figure 4A, a circular proteomic tree of phage MA9V-1 showed that its genome is located in the “others” gray module whatever for “virus family” or “host group.” In addition, we constructed a rectangular proteomic tree (Figure 4B), consists of 11 myovirus genomes, which revealed that the phage MA9V-1 was related to *Sphingomonas* phage PAU (accession no.NC_019521) and was branched together with *Tenacibaculum* phage PTm1 (accession no.NC_049340) and *Tenacibaculum* phage PT24 (accession no.NC_049383) (White and Suttle, 2013; Kawato et al., 2020). Moreover, comparative genomic analysis was performed with genome of phage MA9V-1 and other three similar phages to analyze genomic evolutionary relationship (Figure 4C). The result indicated that MA9V-1 was more closely related to PAU than others, showing a similar region between them mainly located in 7,970–40,762 nt and 172,015–200,768 nt region of MA9V-1 genome with a low similarity (< 50% in most regions), whereas other regions share no similarity with PAU (see Figure 4).

**Figure 5:**
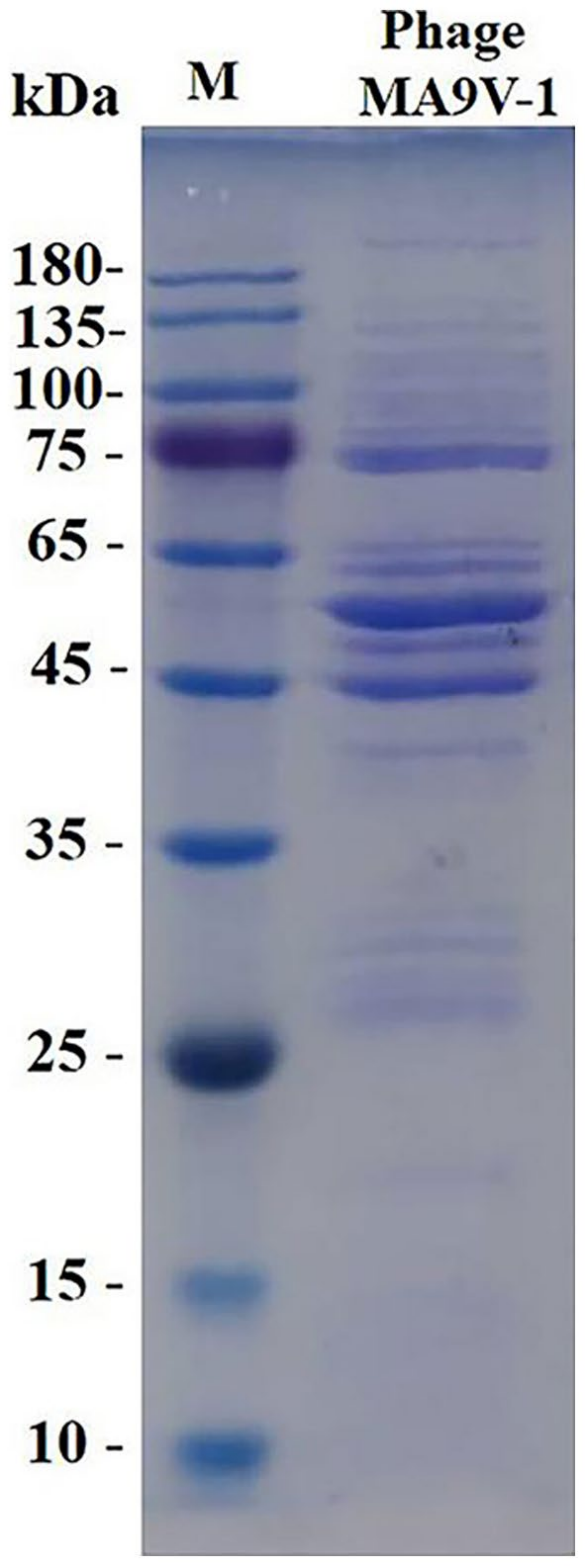
SDS-PAGE analysis of purified phage MA9V-1 virion structural proteins. M: protein ladder with its standard molecular weight on the left.

### 3.5. SDS-PAGE analysis of phage MA9V-1

Structural protein expression analysis of purified phage particles by SDS-PAGE gel electrophoresis showed at least 15 bands of protein molecular weight ranging from 20 to 260 kDa. The protein bands were mainly distributed in 75–135, 45–65, and 25–35 kDa regions of SDS-PAGE gel, which were likely related to structural proteins, terminal family proteins, DNA replication proteins, and hypothetical proteins. According to SDS-PAGE results, we only obtained limited knowledge, and whole-genome analysis of phage MA9V-1 was required to better elucidate the role of all proteins (Lu and Breidt, 2015).

### 3.6. Phylogenetic relationships of phage MA9V-1

**Figure 6:**
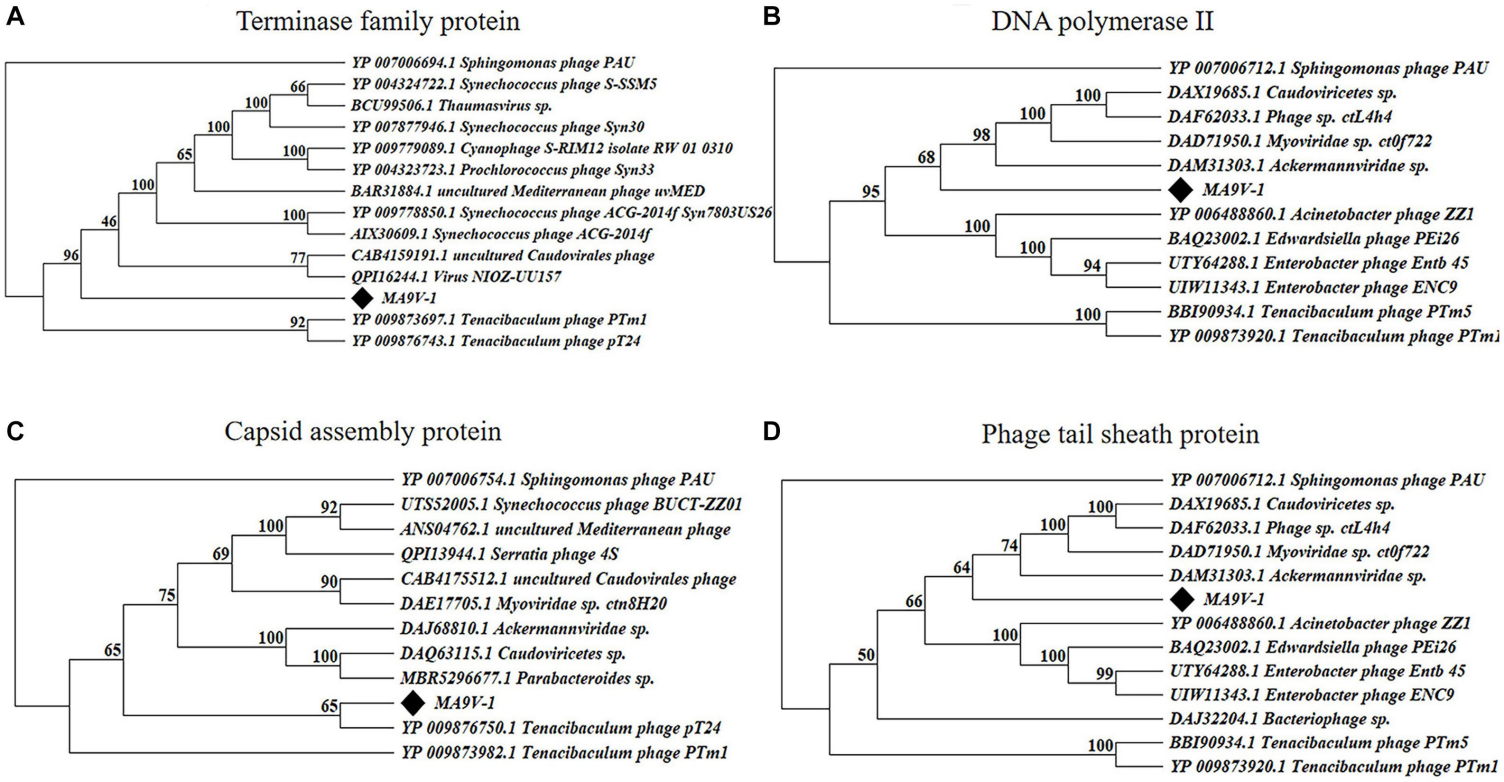
Protein phylogenetic tree of the selected *C. indologenes* phage MA9V-1 and 16 other phages based on deduced amino acid sequences of **(A)** terminase family protein; **(B)** DNA polymerase II; **(C)** capsid assembly protein; and **(D)** phage tail sheath protein. The value on the nodes of the branches indicates the credibility of the tree branch (≥50%). The position of phage MA9V-1 in the picture is marked with a black diamond.

Based on the results of the proteomic tree, we constructed a phylogenetic tree analysis with four representative proteins and aimed to accurately validate the taxonomic classification of phage MA9V-1. Phylogenetic relationships were analyzed among phage MA9V-1 and other 15 members based on the amino acid sequences of four highly conserved proteins, namely terminase family protein (ORF262), DNA polymerase II (ORF135), capsid assembly protein (ORF149), and phage tail sheath protein (ORF001), respectively (Figure 6). The results are presented in Figures 6A,B,D. Phage MA9V-1 was more closely related to *Sphingomonas* phage PAU taking as “tree root” than others and grouped into a clade of an independent branch. Similar tree was also obtained using the deduced amino acid sequences of capsid assembly protein ORF149; we can observe that phage MA9V-1 was branched with *Tenacibaculum* phage pT24, which shared the similar results with above three proteins (Figure 6C). Taken together, phage MA9V-1 is similar to *Sphingomonas* phage PAU, *Tenacibaculum* phage PTm1, and *Tenacibaculum* phage pT24, but there is low identity between them, which was consistent with the conclusion of the proteomic tree analysis, indicating that it is a novel phage against *C. indologenes* MA9. According to the latest guidelines of the International Committee on Classification of Viruses (ICTV),^13^ in March 2021 (Turner et al., 2021), we only concluded that phage MA9V-1 is a new myovirus.

### 3.7. Efficiency of MA9V-1 in phage therapy of *P. notoginseng*

**Figure 7:**
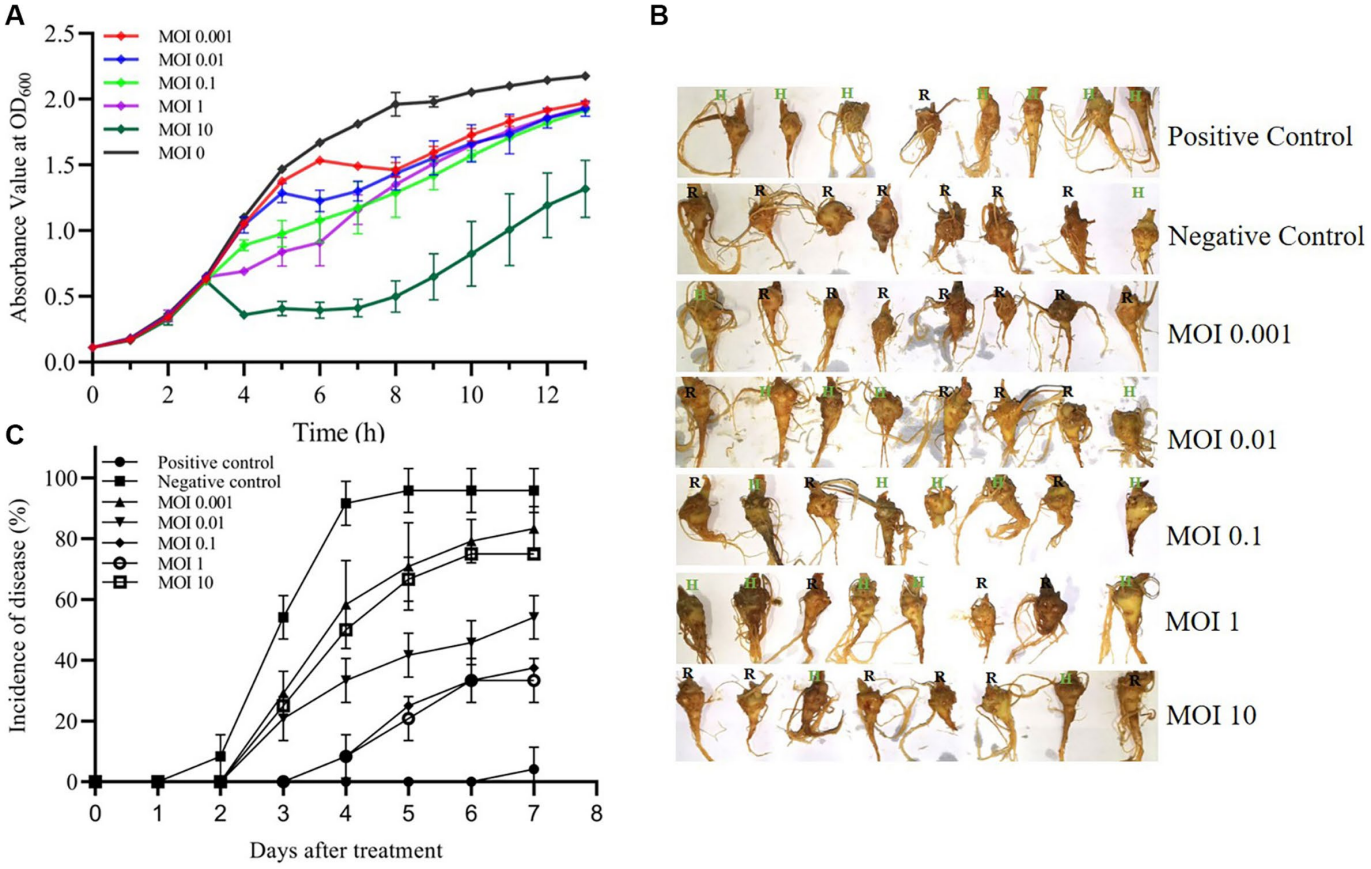
Virulence efficiency of phage MA9V-1 on *C. indologenes* strain MA9. **(A)** The growth inhibitory curve of *C. indologenes* MA9 mixed with phage MA9V-1 at various MOIs of 0 (black), 0.001 (red), 0.01 (blue), 0.1 (green), 1 (purple), and 10 (dark green), respectively. The growth curve was graphed with the absorbance of OD_600_ measured every 60 min for 13 h. **(B)** Representative images of the results after the addition of different mixtures of phage MA9V-1 and *C. indologenes* MA9 to *P. notoginseng* root after treatment for 7 days. **(C)** The curve about the incidence of disease at different MOIs, including 0.001 (regular triangle), 0.01 (inverted triangle), 0.1 (diamond-shaped), 1 (hollow circle), and 10 (hollow square). Positive control (solid circle) was treated with inactive *C. indologenes* MA9, and negative control (solid square) was treated with *C. indologenes* MA9 at the exponential phase. The incidence of disease was calculated as the percentage of rotten roots to the total number of parallel experiments. The bars indicate standard errors.

To investigate the growth inhibitory effect of phage MA9V-1 on the host strain, *C. indologenes* MA9, we first determined the absorbance value at OD_600_ of *C. indologenes* MA9 with and without phage MA9V-1 for 13 h (Figure 7A). In the absence of phage MA9V-1 (MOI = 0), the bacteria reached a stable phase after 8 h of culture, showing a standard “S” growth curve. After adding different titers of phage MA9V-1 into *C. indologenes* MA9 cultures, as shown in Figure 7A, the curve at MOI of 0.001 and 0.01 was not significantly inhibited, until 5 h; the curve showed a downward trend and then regrew relatively rapidly. While using an MOI of 10, an opposite phenomenon to the earlier situation was observed, wherein the bacterial growth sharply reduced and then regrew slowly for 9 h. Meanwhile, the remaining MOIs inhibited the growth for 10 h. Overall, after treatment with phage MA9V-1 at different MOIs, the growth of *C. indologenes* MA9 could be inhibited. Therefore, we took advantage of phage therapy experiments to roughly estimate the therapeutic effectiveness of phage MA9V-1 on *P. notoginseng* root slice infected with *C. indologenes* MA9 for practical purposes (Figure 7B). Among them, the enlargement picture of second sample of MOI = 0.01 and fifth sample of MOI = 10 more intuitively reflects the difference between healthy and rotten samples (Supplementary Figure S1). According to the results shown in Figure 7C, we can observe that exceeded 85% of the root samples of *P. notoginseng* had been rotted in the negative control, which verified the strong pathogenicity of *C. indologenes* MA9. While all groups treated with phage MA9V-1 at different MOIs had samples with only slight or even no symptoms, especially at MOIs of 0.1 and 1, both of them decreased the incidence of disease of *P. notoginseng* root slice up to 50% or more and delayed the occurrence of root rot disease. Preliminary exploratory experiments suggested that the phage MA9V-1 is a good candidate for controlling root rot disease as its inhibition in the development of disease symptoms caused by *C. indologenes* MA9.

## 4. Discussion

Phage MA9V-1, identified and characterized in this study, is capable for infecting the phytopathogenic bacteria *C. indologenes* MA9 causing *P. notoginseng* root rot (Zhang et al., 2020b). The composition of rhizospheric dominant bacterial and fungal genera significantly differed between moderately and severely diseased plants in root rot of *P. notoginseng*, indicating that bacterial pathogens and fungi play critical roles (Dong et al., 2018; Zhang et al., 2020a). However, numerous corresponding studies on this disease rarely focused on bacterial phytopathogens. Furthermore, the rapid development of bacteria resistant to antibiotics and the slow development of new antibiotics greatly stimulated people to seek alternative methods (Kortright et al., 2019). Therefore, applying phage therapy to the bacterial pathogen *C. indologenes* MA9 is expected to provide a potential strategy for solving or alleviating root rot disease of *P. notoginseng*.

MA9V-1 was isolated from sewage like many phages, such as *Clostridium freundii* phage IME-JL8 (Jia et al., 2020), *Vibrio* phage vB_VpaP_FE11P (Yang M. Y. et al., 2022), and *Pseudomonas aeruginosa* phage PPAY (Niu et al., 2022), indicating that sewage is an abundant reservoir of phages. MA9V-1 exhibited as a tiny plaque with a diameter of approximately 0.5–1.5 mm on the lawn of *C. indologenes* MA9, which was smaller than *Enterococcus faecalis* phage EFap02 and *Vibrio parahaemolyticus* phage vB_VpaP_GHSM17 (approximately 2 mm) (Liang et al., 2022; Liu et al., 2022). Morphologically, *C. indologenes* phage MA9V-1, consisted of an icositetrahedron head, a contractible tail, and a relatively short “neck,” was similar to *T. maritimum* phages PTm1 and PTm5, according to the size of whole structural portions. Interestingly, though all of them are myoviruses, several flexible fiber-like appendages of length of 50–100 nm appendages at the upper region of the head of phages PTM1 and PTM5 are clearly observed through the imaging of TEM, while phage MA9V-1 is not observed (Kawato et al., 2020). Furthermore, 95% phage MA9V-1 particles were able to rapidly adsorb to the surface of the cell membrane within 12 min that was shorter than 25 min of *T. maritimum* phage PTm1 and 15 min of MDR *A. baumannii* phage vABPW7 and exhibited a brief latent period (20 min), which can make phage MA9V-1 quickly release progeny virus in a life cycle (Kawato et al., 2020; Wintachai et al., 2022). Moreover, the highly host-specific phage is one of the key factors to phage therapy applied in antibiotic substitutes, medical agents, and agricultural biocontrol (Luong et al., 2020). Due to this unique feature to phage, phage therapy would not directly affect the density, richness, and viability of other microorganisms living in microecology (Wang et al., 2019; Federici et al., 2021). The growth inhibitory curve indicated that the inhibitory ability of MA9V-1 was little weaker than other phages, such as phage *P. syringae* Eisa9 (Korniienko et al., 2022) and *P. aeruginosa* phage PPAY (Niu et al., 2022). We hypothesized a reasonable explanation that phage MA9V-1 required a long time to complete adsorption, invasion, proliferation, maturation, and lysis, since it has a lower burst size value (only 10 PFU/cell), which made it not easy to inhibit the bacterial growth. In addition, comparative genomic and phylogenetic tree analysis showed that phage MA9V-1 has a low similarity with phage *Sphingomonas* PAU and even a lower similarity with other two phages, indicating that phage MA9V-1 is most likely a novel phage infecting *C. indologenes* MA9 (Kwon et al., 2021). Importantly, bacteriophages against *C. indologenes* MA9 have not reported up to present, so further investigation should be needed to explore the characteristics of phage MA9V-1 and its genome sequence.

Lytic phage (also known as virulent phage) can be used for phage therapy of bacterial pathogens because they can continuously complete the life cycle of adsorption, invasion, reproduction, assembling, and release (North and Brown, 2021). Thus, we could derive reasonable inferences about its entire infection process in combination with the predicted functional proteins in the genome. First, to infect, there are proteins relating to tail structure, tail fiber, and others, such as ORF16 short tail fiber protein (Table 2). Then, the tail sheath protein (corresponding to ORF01) of phage MA9V-1 is used like a syringe to inject the genetic content into the host cell, where MA9V-1 utilizes its self-expression protein, including ORF89 DNA helicase, ORF135 DNA polymerase, and ORF95 NAD-dependent DNA ligase to replicate, transcribe, and translate the genetic materials and necessary virion components. Subsequently, recombinase existing in the genome of phage MA9V-1 is used to assemble all the components synthesized in host cells into new progeny phages. Finally, with the function of holin and endolysin proteins (similar to ORF144 Lysozyme RrrD protein), called the lysis system “holin–lysin,” new progeny phages are released to complete the whole process of invasion to host cells (Stone et al., 2019). Additionally, in response to the phage infection, the hosts have evolved a restriction–modification system (R-M), which restricts the ability of endonucleases to rapidly degrade unmethylated phage DNA to prevent phage invasion. Phages have evolved the ability to express methylase to protect their DNA from degradation against the R-M system. For example, the *Bacillus* phage SPR encodes a methyltransferase that modifies three base sites to make phage DNA escape from degradation by multiple nucleases (Murphy et al., 2013). Furthermore, mechanisms of preventing adsorption and CRISPR-Cas adaptive immunity are anti-invasion strategies evolved to fight phages (Hampton et al., 2020). Thus, we could assume reasonably that the efficiency of phage therapy depends mainly on whether the phage can release a large number of progeny phages within a brief time and the rate at which the host cell develops resistance to the phage, combined with the understanding of infection strategies and evolutionary mechanisms between bacteria and phages, which is expected to improve the efficiency of phage therapy.

In summary, the phage MA9V-1 is a novel lytic phage specific to *C. indologenes* MA9, which has a potential preventive and biocontrol effect on *P. notoginseng* root rot caused by *C. indologenes* MA9. However, there are some limitations exposed in this study. For example, treating methods and culturing conditions were both conducted under the laboratory environment, which did not really simulate the microecology surroundings in which microorganisms interact with the rhizospheric soils of *P. notoginseng*, leading to deviations in the therapeutic results. When phage therapy applied in the agricultural environment, its functional mechanism is a complicated process. A reasonable insight is that the host undergoes self-evolutionary in response to phage infection, which leads to modification in the cell membrane structure, making it lose the ability to compete with other microbial populations. The weaker ability to compete for survival resources results in a reduction in the abundance of pathogenic bacterium, which enriched bacterial taxa that are highly antagonistic toward the pathogen and achieved the purpose of phage therapy (Wang et al., 2019). Not only that, whether the stability and efficacy of phage therapy will be affected when it is applied under different environmental conditions, and whether it will destroy the pharmacological components contained in *Panax notoginseng* will become the focus of our future research. Similarly, the application of phage therapy in agriculture also brought many challenges and limitations. One is that the large-scale preparation of phage will cost immense manpower and financial resources, which greatly limits the scale of phage therapeutic application. Another one is whether the residual phages remaining in the soil will cause permanent pollution to soil-like pesticides and antibiotics. Therefore, the wide application of phage therapy is a long and difficult process, and there are still many problems waiting for us to explore and solve. We believe that these experimental data would provide a theoretical basis for controlling the root rot disease of *P. notoginseng* and a new idea for preventing the diseases of traditional medicinal plants, such as *P. ginseng*, *P. quinquefolius*, and *P. japonicus*.

## Data availability statement

The datasets presented in this study can be found in online repositories. The names of the repository/repositories and accession number(s) can be found in the article/Supplementary material.

## Author contributions

HZ wrote the first draft of the manuscript and carried out experiments. YW defined the research theme and revised the manuscript. GJ, JZ, XC and YY provided experimental materials. HZ, YD, JS, JL, and CM designed the methods and experiments and analyzed the results and data. All authors contributed to the article and approved the submitted version.

## Footnotes

^1^ https://sourceforge.net/projects/bbmap/

^2^ https://github.com/ablab/spades

^3^ https://github.com/enormandeau/ncbi_blast_tutorial

^4^ https://proksee.ca/

^5^ https://toolkit.tuebingen.mpg.de/tools/hhpred

^6^ https://www.mgc.ac.cn/VFs/

^7^ https://card.mcmaster.ca/

^8^ https://trna.ucsc.edu/tRNAscan-SE/

^9^ https://www.clustal.org/download/current/

^10^ https://mybiosoftware.com/easyfig-genome-comparison-visualizer.html

^11^ https://www.megasoftware.net/

^12^ https://www.genome.jp/viptree/

^13^ https://ictv.global/

## Supplementary material

Supplementary Figure S1 The image on the left shows the result of the root of *P. notoginseng* after treatment for five days at MOI of 0.01 (Corresponding to 2nd sample of MOI = 0.01); the picture on the right shows the result of the root of *P. notoginseng* after treatment for seven days under the condition at MOI = 10 (Corresponding to 5th sample of MOI = 10).
