# Supplementary figures and images for: Isolation, characterization, and genomic analysis of a novel bacteriophage MA9V-1 infecting Chryseobacterium indologenes: a pathogen of Panax notoginseng root rot
Source: Front Microbiol. 2023 Sep 14;14:1251211. doi: 10.3389/fmicb.2023.1251211 (PMC10537231; doi:10.3389/fmicb.2023.1251211)

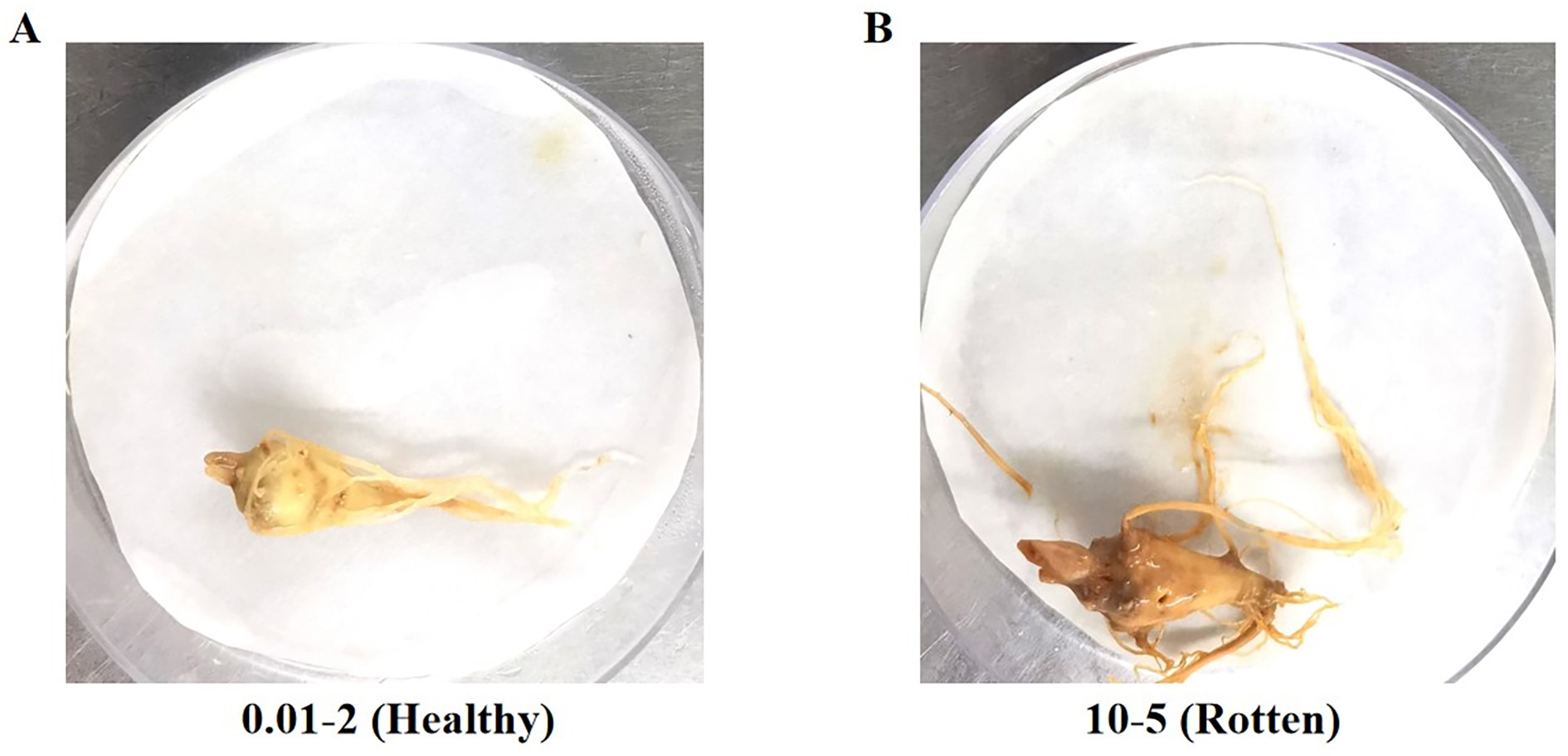

Supplement: Supplementary Figure S1 — The image on the left shows the result of the root of P. notoginseng after treatment for five days at MOI of 0.01 (Corresponding to 2nd sample of MOI = 0.01); the picture on the right shows the result of the root of P. notoginseng after treatment for seven days under the condition at MOI = 10 (Corresponding to 5th sample of MOI = 10). [file Image_1.jpg]
